# NME3 Regulates Mitochondria to Reduce ROS-Mediated Genome Instability

**DOI:** 10.3390/ijms21145048

**Published:** 2020-07-17

**Authors:** Chih-Wei Chen, Ning Tsao, Wei Zhang, Zee-Fen Chang

**Affiliations:** 1 Institute of Molecular Medicine, College of Medicine, National Taiwan University, Taipei 10002, Taiwan; m9605008@gmail.com; 2 Institute of Biochemistry and Molecular Biology, College of Medicine, National Taiwan University, Taipei 10002, Taiwan; oldmagicgod@hotmail.com; 3 State Key Laboratory of Quality Research in Chinese Medicines, Macau University of Science and Technology, Taipa, Macau 999078, China; wzhang@must.edu.mo; 4 Center of Precision Medicine, College of Medicine, National Taiwan University, Taipei 10002, Taiwan; 5 Genomics Research Center, Academia Sinica, Taipei 11529, Taiwan

**Keywords:** NME3, DNA damage, mitochondrial morphology, oxidative stress

## Abstract

NME3 is a member of the nucleoside diphosphate kinase (NDPK) family that binds to the mitochondrial outer membrane to stimulate mitochondrial fusion. In this study, we showed that NME3 knockdown delayed DNA repair without reducing the cellular levels of nucleotide triphosphates. Further analyses revealed that NME3 knockdown increased fragmentation of mitochondria, which in turn led to mitochondrial oxidative stress-mediated DNA single-strand breaks (SSBs) in nuclear DNA. Re-expression of wild-type NME3 or inhibition of mitochondrial fission markedly reduced SSBs and facilitated DNA repair in NME3 knockdown cells, while expression of N-terminal deleted mutant defective in mitochondrial binding had no rescue effect. We further showed that disruption of mitochondrial fusion by knockdown of NME4 or MFN1 also caused mitochondrial oxidative stress-mediated genome instability. In conclusion, the contribution of NME3 to redox-regulated genome stability lies in its function in mitochondrial fusion.

## 1. Introduction

NMEs, nucleoside diphosphate kinases (NDP kinases, NDPK), are a family of highly conserved proteins in eukaryotes [1]. NMEs catalyze the reactions that transfer the terminal phosphate of a nucleoside triphosphate to a nucleoside diphosphate to equilibrate the NDP and NTP pools in a cell [2]. NME1-10 in humans is encoded by ten different genes [1], which are divided into two distinct groups. The group I genes encode proteins that are conserved in vertebrate species and possess the classic enzymatic activity of NDP kinase. This group includes NME1-4 with 58 to 88% identity, in which NME4 is exclusively located in the intermembrane space of mitochondria. Group II includes NME5-9 that are more divergent in the sequences with 22 to 44% identity [3]. 

Up-to-date, NMEs have been shown to participate in a variety of cellular processes, including development, signal transduction, metabolism, and cancer metastasis. We have recently demonstrated that a fatal mitochondrial neurodegenerative disorder is associated with NME3 deficiency due to homozygous mutation at the initiation codon of NME3. The fibroblasts derived from the patient were used to reveal that NME3 is critical for mitochondrial fusion independent of its catalytic function. Intriguingly, the oligomerization and NDPK activity of NME3 is separately required for mitochondrial elongation and ATP formation to support cell survival under conditions of glucose deprivation [4]. 

Mitochondria are dynamic organelles that undergo constantly fusion, fission, transport, and degradation [5,6]. It is well known that mitofusin 1/2 (MFN1/2) are the GTPases mediating outer membrane fusion, while OPA1 is the GTPase at intermembrane space for inner membrane fusion. It has been shown that NME4, located at the inner mitochondrial membrane, interacts with OPA1 to stimulate mitochondria fusion by increasing GTP loading [7]. Although NME3, located on the outer membrane of mitochondria, is capable of forming a complex with MFN1/2, the catalytic function of NME3 is not involved in MFN1/2-mediated fusion [4]. Thus, NME3 and NME4 participate in the regulation of mitochondrial dynamics via different mechanisms. 

By analysis of a human cancer database, it has been shown that low expression of NME3 is correlated with poorer prognosis in a variety of cancers [8]. Given genome instability is a hallmark of cancer [9], we further studied the function of NME3 in genome stability. Our previous work has demonstrated that NME3 can interact with Tip60, a histone acetyltransferase for chromatin remodeling at DNA damage sites [10], to facilitate DNA repair in serum-deprived cells that are deficient of four dNTPs [11]. In contrast, in proliferating cells containing high levels of dNTPs, disruption of the interaction of NME3 with Tip60 has no effect on DNA repair. We proposed that the interaction of NME3 with Tip60 is not necessarily important in damage site-specific supply of dNTPs unless the cells are deficient of dNTPs. In this study, we found that in proliferating cells, knockdown of NME3 delays DNA repair without affecting cellular levels of dNTPs/rNTPs. Our mechanistic investigation demonstrates the role of NME3 in reducing ROS-induced genome instability via its function in mitochondrial fusion. 

## 2. Results

### 2.1. NME3 Knockdown Delays the Repair of DNA Double-Strand Breaks without Affecting Nucleotide Pools

**Figure 1:**
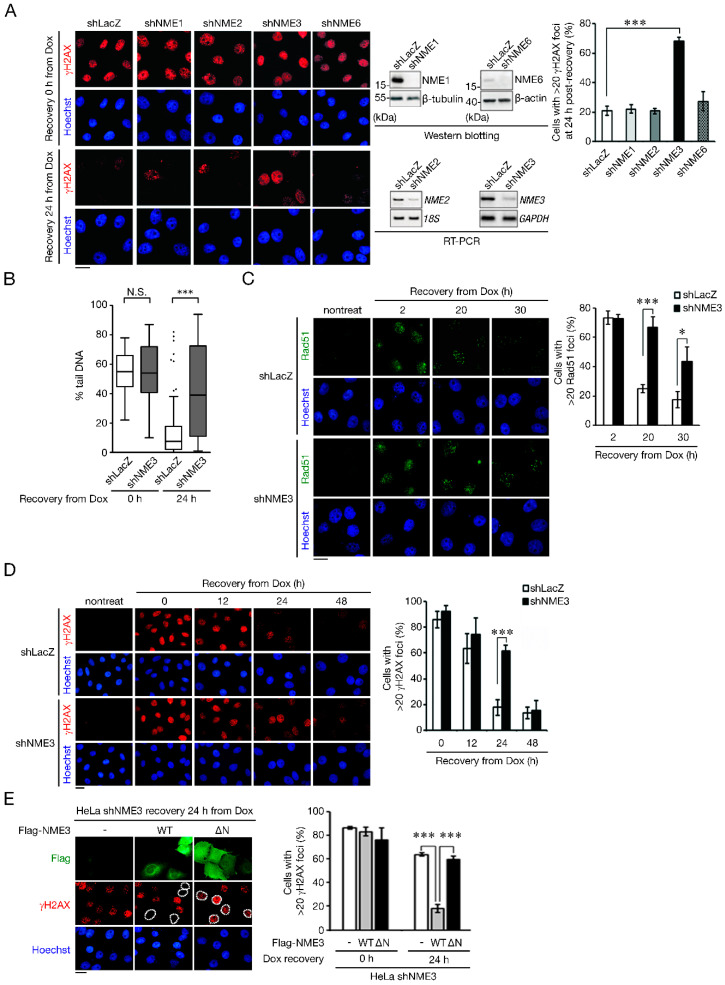
NME3 is required for repairing DNA double-strand breaks. (**A**) HeLa cells were infected with LacZ, NME1, NME2, NME3, or NME6 shRNA lentivirus. After infection for 72 h, cells were treated with doxorubicin (Dox, 1 μM) for 1 h. Cells were recovered in fresh medium for γH2AX immunofluorescence (IF) staining at the time point indicated. The representative images of γH2AX staining (Left). (Scale bar, 20 μm.) Western blotting to indicate NME1 and 6 knockdown and RT-PCR for NME2 and 3 knockdown (Middle). The quantitation results for the percentage of cell with >20 γH2AX foci after recovery 24 h from Dox exposure (Right). Error bar represents s.d. 100 cells were analyzed in each experiment (*n* = 3). (**B**–**D**) HeLa cells infected with lentivirus expressing shRNA of LacZ (shLacZ) and NME3 shRNA (shNME3) were treated with Dox (1 μM) for 1 h, and then recovered at the indicated time. (**B**) Neutral comet assay. The percentage of tail DNA is shown as Box-and-Whisker box plot. More than 50 cells were analyzed in each experiment (*n* = 3). (**C**) IF staining of Rad51 (**D**) and γH2AX Data are shown as mean ± s.d. (100 cells in each experiment, *n* = 3). (Scale bar, 20 μm.) (**E**) HeLa shNME3 cells were transfected with an empty vector, wild-type (WT), or N-terminus deleted mutant (ΔN) of Flag-NME3. These cells were treated with Dox and recovered as described above for IF staining of Flag and γH2AX. Representative images are shown at the left, and the outlines indicate the nuclear boundaries of Flag-positive cells. (Scale bar, 20 μm.) Data are presented as mean ± s.d. 50 cells were analyzed in each experiment (*n* = 3). *** *p* < 0.005, * *p* < 0.05 based on Student’s *t* test. N.S. means no significant differences.

To access the importance of NME3 in genome stability, NME3 was depleted in proliferating HeLa to test its requirement for DNA repair. For comparison, NME1, NME2, and NME6 were also depleted. These cells were treated with doxorubicin, an inhibitor of topoisomerase II, to generate DNA double-strand breaks (DSBs) [12], which were indicated by γH2AX immunofluorescence staining [13]. After 1 h, doxorubicin was washed out and cells were recovered in fresh medium. The intensity of γH2AX foci was similar in control and NME knockdown cells after doxorubicin exposure. At 24 h after recovery, γH2AX foci were already diminished in control, NME1, NME2, and NME6 knockdown cells, indicating the repair of DSB lesions. In contrast, NME3 knockdown cells retained γH2AX foci at 24 h, indicating the impairment of repair (Figure 1A). The neutral comet assay confirmed that DNA lesions remained in NME3 knockdown but not in control after 24 h recovery from doxorubicin treatment (Figure 1B). The major mechanism for repairing DSBs in non-G0/G1 cells is homologous recombination (HR) [14]. We further analyzed Rad51 foci, which indicate strand invasion in HR repair [15]. The result showed that NME3 knockdown did not affect the amount of Rad51 foci at 2 h recovery from doxorubicin exposure (Figure 1C). After 20 h and 30 h, the number of Rad51 foci was significantly reduced in the control cells while sustained in NME3 knockdown cells. This indicates that NME3 knockdown slowed down the resolution of recombinogenic lesions. We prolonged the recovery time to 48 h for the analysis of γH2AX immunofluorescence (IF) staining and found similar levels of γH2AX in shLacZ control and NME3 knockdown cells, confirming that repair of doxorubicin damage also occurred in the latter, albeit delayed (Figure 1D). We have previously shown that the deletion of N-terminal 30 amino-acids causes NME3 to be defective in mitochondrial localization [4]. We further compared the effect of re-expression of Flag-tagged (Flag-NME3), wild-type (WT), or N-terminally truncated mutant (ΔN) in shNME3 cells on repair of doxorubicin-induced DSBs. The results showed that γH2AX foci disappeared in shNME3 cells expressing Flag-NME3 (WT) but not in Flag-NME3 (ΔN)-positive cells (Figure 1E). Thus, N-terminus-mediated mitochondrial localization of NME3 affects DNA repair. 

**Figure 2:**
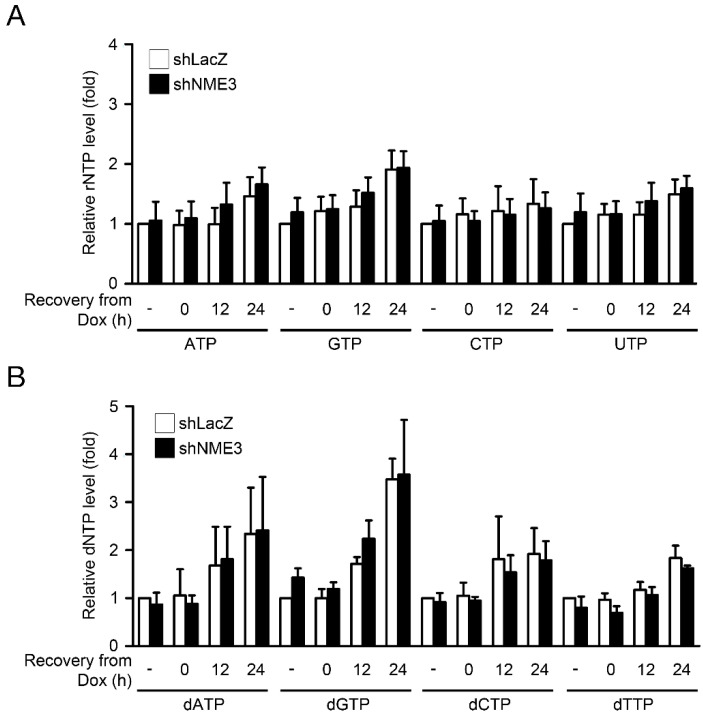
Effect of NME3 knockdown on NTP pools. HeLa cells were infected with lentivirus harboring shRNA against LacZ or NME3. After infection for 72 h, cells treated with doxorubicin (Dox, 1 μM) for 1 h and recovered at the indicated time were harvested for liquid chromatography-tandem mass spectrometry (LC/MS/MS) analysis for determining cellular levels of rNTP and dNTP pools. The levels of (**A**) rNTP and (**B**) dNTP relative to untreated cells (-) are shown as the mean ± s.d. (*n* = 3).

We further analyzed the effect of NME3 knockdown on NTP pools by liquid chromatography-tandem mass spectrometry (LC/MS/MS) analysis in HeLa cells [16]. The results showed that both rNTP and dNTP pools were not affected by knockdown of NME3, indicating that NME3 does not contribute to steady-state levels of NTPs (Figure 2). In parallel, cells were treated with doxorubicin and recovered at different time points for analysis of NTP pools. We found that ATP, GTP, and four dNTP pools were elevated in these cells after recovery from doxorubicin exposure, and NME3 knockdown did not affect this elevation during DNA repair. These results suggest that knockdown of NME3 suppresses DNA repair without affecting total cellular NTP pools. 

### 2.2. NME3 Knockdown Increases SSBs and Suppresses DNA Repair via Mitochondrial Oxidative Stress

**Figure 3:**
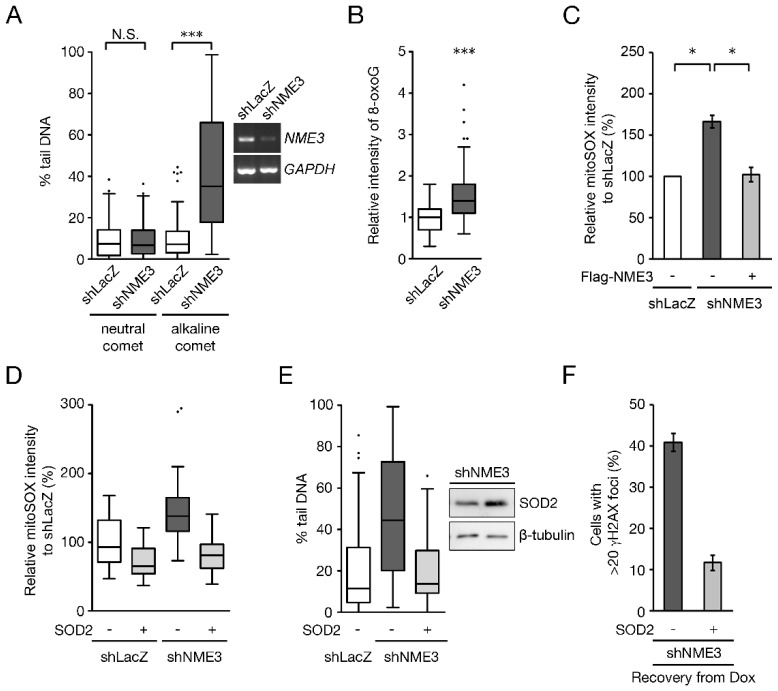
Knockdown of NME3 increases genome instability by mitochondrial oxidative stress. HeLa cells infected with lentivirus expressing shRNA of LacZ (shLacZ) or NME3 (shNME3) were subjected to neutral and alkaline comet assay (**A**), and IF staining of 8-oxo-guanosine (**B**). The quantitative result for the percentage of tail DNA is shown in a Box-and-Whisker box plot (Tukey). NME3 knockdown was confirmed by RT-PCR. More than 180 cells were analyzed for the percentage of tail DNA (*n* = 3). *** *p* < 0.005 based on Student’s *t* test. N.S. means no significance. (**B**) The intensity of 8-oxo-guanosine (8-oxoG) was quantitated in 180 cells (*n* = 3). *** *p* < 0.005. (**C**) HeLa cells expressing shLacZ and shNME3 were infected with a lentivirus of Tet-on Flag-NME3. After doxycycline (4 μg/mL) induction for 16 h, cells were stained with MitoSOX and analyzed by flow cytometry. The relative intensity of MitoSOX was expressed as mean ± SEM (*n* = 3). * *p* < 0.05. (**D**,**E**) HeLa cells expressing shLacZ and shNME3 were infected with an expression vector of SOD2 for 2 days, followed by MitoSOX staining (**D**) and alkaline comet analysis (**E**). The fluorescence intensity of MitoSOX was captured by a fluorescence microscopy (AxioObserver A1, Carl Zeiss) with AxioVision software (v4.8, Carl Zeiss). More than 45 cells were analyzed for the intensity of MitoSOX. (**E**) The expression of SOD2 was confirmed with Western blotting and was applied for alkaline comet analysis. More than 80 cells were counted. (**F**) Cells were treated with doxorubicin (1 μM) for 1 h and then recovered for 21 h for IF staining of γH2AX (*n* = 2).

We then asked the question of whether NME3 knockdown increases the extent of doxorubicin-induced DNA breaks and thus requires longer periods for repair. Control and NME3 knockdown cells without doxorubicin treatment were subjected to neutral and alkaline comet assays, in which the former detects DSBs, and the latter single-strand breaks (SSBs), together with DSBs and alkali labile sites. NME3 knockdown increased the percentage of tail DNA in alkaline but not in the neutral comet assay, indicating that NME3 knockdown specifically increases SSBs or alkali-labile sites (Figure 3A). ROS-mediated DNA damage is the major source of SSBs [17]. Next, we tested the effect of NME3 knockdown on oxidative stress. Data of immunofluorescence staining of 8-oxo-guanosine (8-oxoG) indicated that NME3 knockdown increased the cellular level of oxidative stress (Figure 3B). MitoSOX measurement showed that NME3 knockdown increased mitochondrial oxidative stress (Figure 3C). We further asked whether the increases in SSBs by NME3 knockdown are the results of mitochondrial oxidative stress. To address this question, mitochondrial superoxide dismutase (SOD2) was overexpressed in NME3 knockdown cells to reduce mitochondrial oxidative stress for alkali comet assay. MitoSOX staining confirmed the effect of SOD2 overexpression on reducing mitochondrial oxidative stress (Figure 3D), and the alkali comet results showed the decrease in the percentage of tail DNA by SOD2 overexpression in these cells (Figure 3E). Consistently, SOD2 overexpression also ameliorated the repair of doxorubicin-induced DSBs in NME3 knockdown cells as revealed by reduction in γH2AX IF staining (Figure 3F). Thus, the function of NME3 in the maintenance of genome stability is through the regulation of mitochondrial oxidative stress. 

### 2.3. NME3-Regulated Mitochondrial Elongation Correlates with Genome Stability

**Figure 4:**
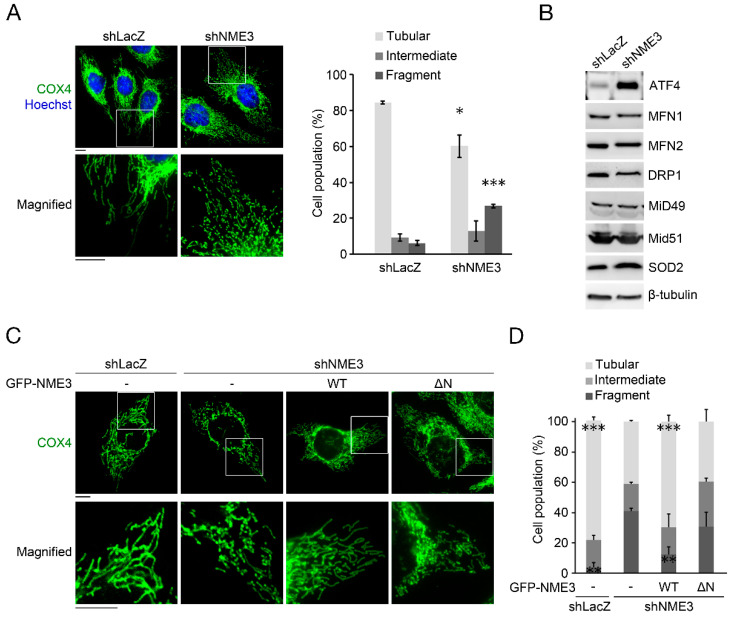
The critical role of NME3 in maintaining mitochondrial morphology. (**A**) Representative images of mitochondrial morphology of HeLa cells expressing shLacZ or shNME3 are shown. Cells were fixed for COX4 IF staining as a mitochondrial marker. (Scale bar, 10 μm.) Cells that contain >75% of total mitochondria in tubular or fragmented organization were categorized and the rest of the population was defined as cells with mitochondria of intermediate morphology. Mitochondrial morphology in tubular, intermediate, and fragmented were counted from 100 cells (*n* = 3). Data are presented as mean ± SEM. * *p* < 0.05, *** *p* < 0.005 compared with that of shLacZ by Student’s *t* test. (**B**) HeLa cells expressing shLacZ or shNME3 were harvested for Western blotting using antibodies against ATF4, MFN1/2, DRP1, Mid49/51, SOD2, and β-tubulin. (**C**,**D**) shLacZ and shNME3 cells were transfected with GFP-NME3 variants including wild-type (WT) and N-terminal deleted-mutant (ΔN) for 8 h and then fixed for mitochondrial morphology analysis. (**C**) Representative images of COX4 staining. (Scale bar, 10 μm.) (**D**) Quantitation results of mitochondrial morphology were analyzed from 100 cells (*n* = 3). Data are presented as mean ± SEM. ** *p* < 0.01, *** *p* < 0.005 compared with that of shNME3 based on Student’s *t* test.

We have previously demonstrated that NME3 knockdown markedly slowed down mitochondrial fusion in human BJ fibroblasts [4]. Fusion makes mitochondria assembled into a tubular network, while fission makes mitochondria more fragmented [18]. The mitochondrial morphology analysis by COX4 staining, a mitochondrial marker, showed that NME3 knockdown increased mitochondrial fragmentation in HeLa cells (Figure 4A). MFN1/2, DRP1, and Mid49/51 are involved in mitochondrial fusion and fission [5]. We found that the cellular levels of these proteins remained unaffected by NME3 knockdown (Figure 4B), while the level of ATF4, an indicator of mitochondrial stress [19] and mitochondrial superoxide dismutase (SOD2), was increased. Re-expression of wild-type NME3 restored mitochondrial tubular network (Figure 4C,D). As expected, the expression of N-terminal deleted-mutant (ΔN) of NME3 failed to restore mitochondrial elongation network (Figure 4C,D), confirming that NME3 localization on the mitochondrial outer membrane is required for mitochondrial elongation. 

**Figure 5:**
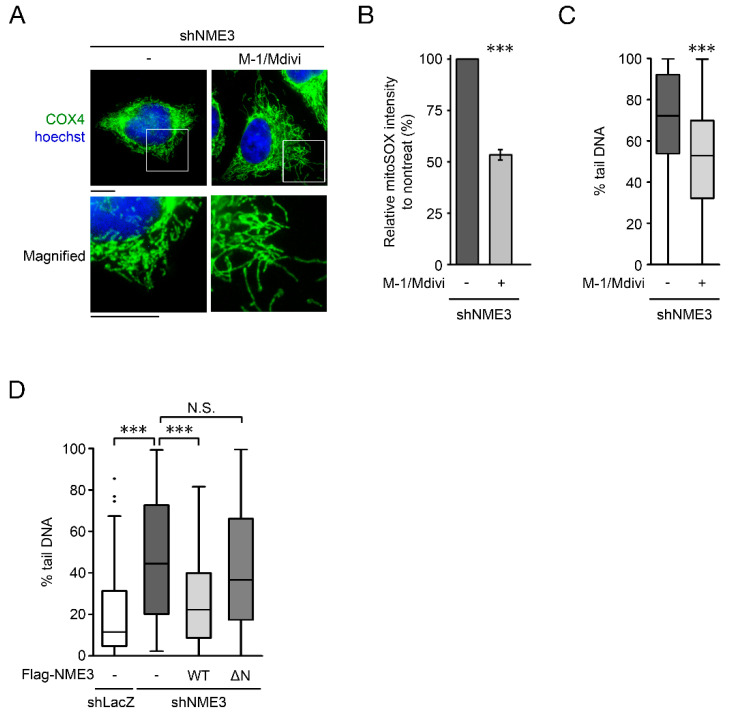
NME3 regulates genome stability via mitochondrial dynamics. HeLa cells infected with shNME3 were treated with M-1 (10 μM) and Mdivi (20 μM) for 3 days and then fixed for analysis. (**A**) Evaluation of mitochondrial morphology by COX4 IF staining. Representative images of mitochondrial morphology of HeLa shNME3 cells treated with M-1/Mdivi are shown (Scale bar, 10 μm). (**B**) Relative levels of mitochondrial oxidative stress by MitoSOX staining were determined by flow cytometry (*n* = 3). Data are presented as mean ± SEM. *** *p* < 0.005 by Student’s *t* test. (**C**) Alkaline comet assay. Quantitation results of the percentage of tail DNA from 100 cells are expressed in a box plot. *** *p* < 0.005. (**D**) HeLa shLacZ and shNME3 cells were infected with a lentivirus of Tet-on Flag-NME3, including wild-type (WT) and N-terminal deleted-mutant (ΔN). After doxycycline (4 μg/mL) treatment for 72 h to induce the expression, these cells were subjected to alkaline comet analysis. The percentage of tail DNA in 100 cells was quantitated from three independent experiments (*n* = 3). *** *p* < 0.005. N.S. represents no significance.

To understand the link between NME3-regulated mitochondrial dynamics and genome stability, we then treated these NME3 knockdown cells with M-1/Mdivi, which are fusion promoter and fission inhibitor, respectively [20,21]. The treatment rescued mitochondrial tubular morphology and significantly reduced mitochondrial oxidative stress (Figure 5A,B). Importantly, the comet assay indicated that this co-treatment also diminished SSBs in NME3 knockdown cells (Figure 5C). We further expressed WT and ΔN mutant of NME3. Consistently, WT but not ΔN mutant of NME3, prevented NME3 knockdown-induced SSBs (Figure 5D). This finding is in agreement with aforementioned results that the repair of doxorubicin-induced DSBs in NME3 knockdown cells was restored by expression of WT but not ΔN mutant of NME3 (Figure 1E). Altogether, these data suggest that it is the function of NME3 in mitochondrial fusion that impacts genome stability. 

### 2.4. Disruption of Mitochondrial Fusion Generally Causes Genome Instability

**Figure 6:**
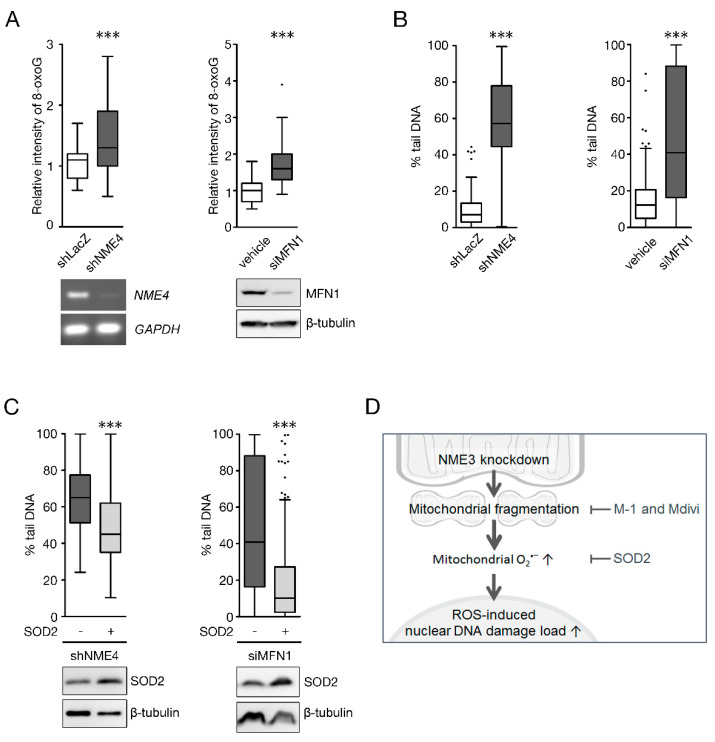
Mitochondrial fusion deficiency induces nucleotide oxidation and mitochondrial ROS-dependent genome lesions. HeLa cells infected with lentivirus of shLacZ, shNME4 or transfected with siRNA against MFN1 (siMFN1) for 2 days were subjected to IF staining of 8-oxoG (**A**) and alkaline comet assay (**B**). (**A**) The quantitative results of 8-oxoG intensity are shown (55 cells for shNME4 set (*n* = 2) and 75 cells for siMFN1 set (*n* = 3)). *** *p* < 0.005 based on Student’s *t*-test. Knockdown of NME4 and MFN1 were confirmed by RT-PCR and Western blotting, respectively. (**B**) Alkaline comet assay. The results of the percentage of tail DNA were determined from 70–180 cells (*n* = 2 for shNME4 and *n* = 3 for siMFN1 set). (**C**) shLacZ, shNME4, and siMFN1 cells were infected with an expression vector of SOD2 for 2 days followed by alkaline comet analysis. The results of the percentage of tail DNA were determined from 88–180 cells (*n* = 2 for shNME4 and *n* = 3 for siMFN1 set). (**D**) The proposed model. NME3 is essential for maintaining mitochondrial elongated morphology in proliferating cells. The loss of NME3 promotes mitochondrial fragmentation, by which mitochondrial oxidative stress and nucleotide oxidation are increased to cause nuclear genome lesions.

To confirm the general importance of mitochondrial fusion in genome stability, we further targeted other molecules involved in the fusion process. NME4 is the mitochondrial NDPK located in the intermembrane space and critical for OPA1-mediated fusion of the inner mitochondrial membrane [7], whereas MFN1 is a GTPase in outer membrane fusion. We then depleted MFN1 or NME4 to test whether the loss of mitochondrial fusion increases oxidative stress with DNA SSBs. In agreement, knockdown of NME4 or MFN1 increased the cellular level of 8-oxoguanine (Figure 6A). Similar to the effect of NME3 knockdown, the comet assay showed that knockdown of either NME4 or MFN1 also significantly increased SSBs (Figure 6B), which was abolished by overexpression of SOD2 (Figure 6C). These results led us to propose that the decreased expression of NME3 slows down mitochondrial fusion to increase mitochondrial fragmentation and oxidative stress, in turn accelerating genome instability (Figure 6D). 

## 3. Discussion

The importance of mitochondrial fusion in controlling cellular oxidative stress has been illustrated by MFN1- or MFN2-knockout cells that display fragmented mitochondria with increased levels of ROS [22] and mutation of *Drosophila OPA1* (*dOPA1*) that causes irregular mitochondrial morphology with elevated amounts of ROS [23]. It has been suggested that fragmented mitochondria are poorer in respiratory super-complex assembly for electron transport chain reactions (ETC), thereby increasing electron leakage to generate superoxide production [24,25]. Here, we present evidence that NME3 is critical for promoting the mitochondrial tubular network. Therefore, the loss of NME3 leads to mitochondrial fragmentation, in turn increasing mitochondrial superoxide formation. As a consequence, ROS-induced DNA oxidation then generates DNA SSBs [17]. Since the increased amount of SSBs observed in NME3 knockdown cells was reduced by SOD2 expression or inhibition of mitochondrial division, these results support our hypothesis that the increase of fragmented mitochondria by NME3 knockdown causes ROS-mediated genome instability. The delayed repair of doxorubicin-induced DSBs is likely due to the increase in ROS-induced DNA damage loads in NME3 knockdown cells. In support of this notion, re-expression of N-terminal deleted-mutant of NME3 (ΔN) mutant defective in mitochondrial binding was unable to reduce SSBs and the repair of DSBs in NME3 knockdown cells. In line with this scenario, knockdown of other mitochondrial fusion proteins, including MFN1 and NME4, also increases SSBs. It is known that the nuclear genome is under constant threats from the byproducts of metabolic reactions [26]. Our data point out a notion that two NDP kinases, NME3 and NME4, coordinate mitochondrial fusion at outer and inner membranes to limit the generation of free radicals from metabolism that impacts genome integrity. Given genome instability as a driver of tumor evolution, the correlation of low NME3 with poorer survival in a number of cancers is closely related to its function in mitochondrial regulation that controls redox to influence genome stability [8]. 

In this study, we found that the cellular levels of four rNTPs and dNTPs were not affected by NME3 knockdown before and after recovery from doxorubicin recovery. This indicates that NME3 does not play a major role in generating rNTPs and dNTPs in the cells, probably because NME3 is much less expressed than NME1/2. One report has shown that NME3 is translocated to the plasma membrane, where its interaction with heterotrimeric G proteins for GTP loading leads to dysregulation of cAMP formation in cardiomyocytes [27]. This indicates the physiological contribution of the local GTP formation by NME3. Our previous study showed that NME3 can interact with Tip60 to increase the local supply of dNTPs in non-proliferating cells that are deficient of dNTPs [11]. Apparently, there are multiple faces of NME3 in a variety of cellular processes via its catalytic function, oligomerization, protein interaction, and subcellular location. In the aspect of genome stability, NME3 in proliferating cells acts by regulating mitochondrial oxidative stress, whereas in non-proliferating cells its function involves local dNTP formation. 

## 4. Materials and Methods

### 4.1. Cell Culture and Transfection

HeLa and HEK293T cells were maintained in Dulbecco’s modified Eagle’s medium (DMEM) (Gibco, Waltham, MA, USA) supplemented with 10% fetal bovine serum (FBS), 100 U/mL penicillin, and 10 μg/mL streptomycin. For lenti-virus package, HEK293T cells were co-transfected with pCMVdeltaR8.91, pCMV, VSVG, and pLKO.1 shRNA plasmids. After transfection for 48 and 72 h, supernatants containing lentivirus were filtered through polyvinylidene difluoride (PVDF) membrane (pore size 0.45 μm, Millipore, Burlington, MA, USA). The siRNA against NME3 was purchased from Sigma-Aldrich (St. Louis, MO, USA. SASI_Hs01_00161811). The siRNA against MFN1 were purchased from Dharmacon (Lafayette, CO, USA. SMARTpool M-010670-01-0005). 

### 4.2. Plasmids and Reagents 

The SOD2 expression vector, pAS3w.SOD2.bsd, was constructed by insertion of PCR products of pBI-EGFP-MnSOD, which was purchased from Addgene (plasmid #16612), at *Nhe*I (5′) and *Pme*I (3′) to pAS3w.bsd vector. Wild-type (WT) and N-terminal 30 amino acids deleted mutant (ΔN) of NME3-GFP expression vectors were constructed by insertion of PCR products amplified from NME3 cDNA at *Xho*I (5′) and *Hind*III (3′) to pEGFP-N1 vector. Tet-on expression vectors of Flag-NME3 were generated as described previously [4]. Mitochondrial Fusion promoter M-1 and Mdivi were purchased from Sigma-Aldrich (SML0629 for M-1, M0199 for Mdivi-1). Antibodies used in this study: γH2AX (Millipore, 05-636), Flag (Sigma-Aldrich, F3165), NME1/nm23-H1 (sc-343, Santa Cruz, Dallas, TX, USA), NME6 (GTX128818, Genetex, Irvine, CA, USA.), 8-oxoG/8-hydroxy-guanosine (ab62623, Abcam, Cambridge, UK), SOD2 (Millipore, 06-984), COX4 (4850S, Cell Signaling, Danvers, MA, USA), ATF4 (Cell Signaling, 11815S), MFN1 (Cell Signaling, 14739S), MFN2 (Abcam, ab56889), DRP1 (Cell Signaling, 8570S), SMCR7/Mid49 (16413-1-AP, Proteintech, Rosemont, IL, USA), SMCR7L/Mid51 (Proteintech, 20164-1-AP), β-tubulin (Sigma-Aldrich, T4026), β-actin (Sigma-Aldrich, A5441).

### 4.3. RT-PCR and Primers 

The RT-PCR were performed as previously described [11]. The primers for *NME2*, *NME3*, *18S* rRNA, and *GAPDH* were used as previous described [11]. For *NME4* detection, the primers (5′-GCGTCCACATCAGCAGGAAT-3′, 5′-GCTGACGGAGGTAGTTGGTC-3′) were used. 

### 4.4. Immunofluorescence (IF) Staining of γH2AX, 8-oxoG, and COX4

Cells were fixed with 4% paraformaldehyde at 37 °C for IF staining of γH2AX, Flag, 8-oxoG, and COX4. The fixed cells on glass were permeabilized with Tris-buffered saline containing 0.3% triton x-100 (TBST) for 10 min followed by blocking with TBST containing 3% bovine serum albumin and 5.5% of normal goat serum (16210064, ThermoFisher Scientific, Waltham, MA, USA) for 1 h. Afterwards, cells were incubated with TBST containing primary antibodies against γH2AX, Flag, 8-oxoG, or COX4 overnight at 4 °C. After three times of TBST wash, cells were incubated with FITC- or TRITC-conjugated secondary antibodies with Hoechst 33342 for DNA staining for 1 h, followed by TBST washing and slide mounting. The images of γH2AX, Flag and 8-oxoG were acquired by Carl Zeiss fluorescence microscope with an AxioCam digital camera, and the intensities were analyzed by AxioVision Rel.4.8 imaging software (Carl Zeiss, Oberkochen, Germany). The intensity of 8-oxoG in each cell were normalized with background value of non-cell area. The images of mitochondria morphology by COX4 staining were acquired by using a confocal fluorescence microscopy (LSM780, Carl Zeiss) equipped with ZEN software (Carl Zeiss, v2009) or a fluorescence microscopy (AxioObserver A1, Carl Zeiss) with AxioVision software (v4.8, Carl Zeiss). 

### 4.5. DNA Comet Analysis 

Alkaline and neutral comet assays were performed using a reagent kit of Single-Cell Gel Electrophoresis Assay kit (4250-050-k, Trevigen, Gaithersburg, MD, USA). Image data were analyzed by CometScore software (v1.6, TriTek Corp, Sumerduck, VA, USA) for the measurement of percentage of tail DNA. 

### 4.6. The Measurement of NTP Pools 

The sample preparation procedures were as described [16]. Briefly, 2 × 10^6^ cells were harvested. Cell pellets were treated with 150 μL of 15% trichloroacetic acid. The acidic supernatants were separated and neutralized twice with 80 μL mixture of trioctylamine and 1,1,2-trichlorotrifluoroethane in a ratio of 45:55 (*v*/*v*). Finally, samples were dried by vacuum pump and store at −80 °C for LC/MS/MS analysis. 

### 4.7. The Measurement of Mitochondrial Oxidative Stress

HeLa cells were treated with 3.75 μM of MitoSOX Red reagent (Thermo Fisher Scientific, M36008) for 10 min at 37 °C. Cells were trypsinized and washed with PBS, after which cells were analyzed by flow cytometry (FACS Calibur, BD, Franklin Lakes, NJ, USA) for quantification of relative intensity of MitoSOX Red. For the SOD2 overexpression experiment, the fluorescent intensity of MitoSOX Red were captured with a fluorescence microscopy (AxioObserver A1, Carl Zeiss) with AxioVision software (v4.8, Carl Zeiss). The cellular intensity of MitoSOX in each cell was analyzed with AxioVision software (v4.8, Carl Zeiss) with background value normalization. 

### 4.8. Statistical Analysis 

The Box-and-Whisker (Tukey method) plots were generated by using Prism (v5.01, GraphPad Software). The column graphs were generated by using Excel (Microsoft Office Professional Plus 2013). All values are presented as mean ± standard deviation (s.d.) or standard error of mean (SEM) as indicated. Statistical analysis of the results from more than three independent experiments was performed by Student’s *t*-test (two-tailed). The *p*-value less than 0.05 was considered as statistically significant.
